# Evaluation of transcutaneous near-infrared spectroscopy for early detection of cardiac arrest in an animal model

**DOI:** 10.1038/s41598-023-31637-1

**Published:** 2023-03-20

**Authors:** Katharina Raschdorf, Arman Mohseni, Kaavya Hogle, Amanda Cheung, Kitty So, Neda Manouchehri, Mahsa Khalili, Saud Lingawi, Brian Grunau, Calvin Kuo, Jim Christenson, Babak Shadgan

**Affiliations:** 1 International Collaboration on Repair Discoveries (ICORD), University of British Columbia, 818 West 10th Avenue, Vancouver, BC V5Z 1M9 Canada; 2 Department of Neuroscience, University of British Columbia, 2215 Wesbrook Mall, Vancouver, BC V6T 1Z3 Canada; 3 School of Biomedical Engineering (SBME), University of British Columbia, 2222 Health Sciences Mall, Vancouver, BC V6T 1Z4 Canada; 4 Department of Emergency Medicine, University of British Columbia and St. Paul’s Hospital, Vancouver, BC Canada; 5 Department of Orthopaedics, University of British Columbia, 2775 Laurel Street, Vancouver, BC V5Z 1M9 Canada

**Keywords:** Physiology, Cardiology, Optics and photonics

## Abstract

Sudden cardiac arrest (SCA) is a leading cause of mortality worldwide. The SCA-to-resuscitation interval is a key determinant of patient outcomes, highlighting the clinical need for reliable and timely detection of SCA. Near-infrared spectroscopy (NIRS), a non-invasive optical technique, may have utility for this application. We investigated transcutaneous NIRS as a method to detect pentobarbital-induced changes during cardiac arrest in eight Yucatan miniature pigs. NIRS measurements during cardiac arrest were compared to invasively acquired carotid blood pressure and partial oxygen pressure (PO_2_) of spinal cord tissues. We observed statistically significant decreases in mean arterial pressure (MAP) 64.68 mmHg ± 13.08, *p* < 0.0001), spinal cord PO_2_ (38.16 mmHg ± 20.04, *p* = 0.0028), and NIRS-derived tissue oxygen saturation (TSI%) (14.50% ± 3.80, *p* < 0.0001) from baseline to 5 min after pentobarbital administration. Euthanasia-to-first change in hemodynamics for MAP and TSI (%) were similar [MAP (10.43 ± 4.73 s) vs TSI (%) (12.04 ± 1.85 s), *p* = 0.3714]. No significant difference was detected between NIRS and blood pressure-derived pulse rates during baseline periods (*p* > 0.99) and following pentobarbital administration (*p* = 0.97). Transcutaneous NIRS demonstrated the potential to identify rapid hemodynamic changes due to cardiac arrest in periods similar to invasive indices. We conclude that transcutaneous NIRS monitoring may present a novel, non-invasive approach for SCA detection, which warrants further investigation.

## Introduction

Sudden cardiac arrest (SCA) is a leading cause of mortality worldwide, accounting for 15–20% of all natural deaths across the USA and Western Europe^1,2^. SCA is defined as the sudden cessation of effective ventricular contractions, leading to inadequate cardiac output and hemodynamic collapse^3^. The loss of systemic circulation is fatal if not immediately detected and treated by initiating high-quality resuscitative measures^4^. Resuscitation is generally most successful when performed within five minutes of circulatory collapse^5–7^. Current estimates suggest that only 25% of SCAs are bystander-witnessed. Hence, in most cases, no witnesses are available to provide life-saving treatment or contact emergency medical services (EMS), prolonging the time it takes to initiate resuscitative measures^8^. Consequently, when EMS professionals arrive on the scene, they elect not to attempt resuscitation in approximately 50% of cases, as life-saving measures are deemed futile at this stage^8^. In attempts to reduce time-to-resuscitation intervals, there is a need to develop cost-effective, wearable systems that can noninvasively monitor parameters related to cardiac activity. This has the potential to reduce average time-to-resuscitation intervals through rapid identification of SCA and automatic notification to medical dispatchers. The use of such technology is of particular interest in high-risk populations, including patients with a prior history of cardiac arrest, congenital heart disease or cardiomyopathy^2^.

Near-infrared spectroscopy (NIRS) is a noninvasive optical biosensing technique that can monitor real-time changes in tissue oxygenation and hemodynamics transcutaneously^9–14^. NIRS technology is based on similar physical principles to photoplethysmography (PPG), a technology that has found routine application in clinical practice with the emergence of pulse oximetry^15–18^. PPG sensors employ two light sources (commonly one red/green and one infrared light emitter) to evaluate arterial blood oxygenation by detecting local blood volume changes arising from systolic cardiac activity^19,20^. These pulsatile changes in light absorption are detected by a photodetector and converted into an estimate of the arterial oxygen saturation (SpO_2_) based on the optical path length of the medium and the Beer–Lambert Law^19,21^. One drawback of PPG sensors is that they require pulsatile blood flow to give a reliable estimate of oxygen saturation, which may limit their use in settings where the pulse is weak or absent, such as cardiac arrest^22–24^.

Using principles similar to pulse oximetry, continuous-wave NIRS employs near-infrared light (between 650 to 1000 nm) to penetrate tissue and measure the amount of light absorbed by tissue chromophores (mostly hemoglobin and water)^25^. With the use of multiple source-detector pairs in a spatially-resolved configuration, NIRS can monitor changes in tissue oxygen delivery and consumption by measuring the following parameters: oxyhemoglobin (O_2_Hb), deoxyhemoglobin (HHb), total hemoglobin (THb = O_2_Hb + HHb; a measure of local blood volume changes), and the hemoglobin difference (Hbdiff = O_2_Hb-HHb, an indirect measure of oxygen utilization)]^26^. Furthermore, it is possible to derive an absolute estimate of the local tissue oxygen saturation^26–28^ (tissue saturation index or TSI; expressed in %) that reflects contributions from arterial (25%), capillary (5%), and venous (70%) compartments alike^29^. Importantly, with sampling rates of 10 Hz and above, transcutaneous NIRS sensors can detect and monitor the effect of cardiac contractile activity on tissue hemodynamics similar to PPG-based monitoring; however, unlike PPG-based technologies, pulsatile blood flow is not a prerequisite to obtaining an estimate of tissue oxygenation, which means that NIRS may have better utility in SCA detection and monitoring^12^.

The study aimed to evaluate whether transcutaneous NIRS could detect changes in pulse and tissue oxygenation during pentobarbital-induced cardiac arrest in our porcine model. After IV pentobarbital administration, changes in NIRS-derived tissue saturation index [TSI (%), absolute value] and oxygenated hemoglobin (O_2_Hb, relative value) were continuously monitored. We compared these results to gold-standard invasive blood pressure monitoring and an invasive spinal cord oxygenation monitor. Such information is essential for evaluating NIRS-based monitoring as a candidate for out-of-hospital SCA detection.

## Materials and methods

### Study design and setting

This investigation was done in coordination with a study to evaluate tissue oxygenation in spinal cord contusion-compression injuries^30^. All animal protocols and procedures performed in this study were approved by the Animal Care Committee of the University of British Columbia (UBC) and were compliant with the policies of the Canadian Council of Animal Care, the U.S. Army Medical Research and Material Command (USAMRMC), and Animal Care and Use Review Office (ACURO). The UBC Center for Comparative Medicine established the anesthesia and analgesia protocols. The study complied with ARRIVE guidelines.

### Porcine model

Data from eight female Yucatan miniature pigs (weighing 24–31 kg) were analyzed as part of other NIRS studies investigating spinal cord hemodynamics after spinal cord injury (SCI). Animals were prepared for surgery, intubated and anesthetized as previously described^31,32^. Animals were pre-medicated with intramuscular Telazol (4–6 mg/kg), xylazine (1 mg/kg), and atropine (0.02–0.04 mg/kg). For anesthesia induction, propofol (2 mg/kg) or isoflurane (2–3% in O_2_) was used before the animals underwent endotracheal intubation. Propofol (8 mg/kg/h), fentanyl (12 µg/kg/h), ketamine (11 mg/kg/h) and midazolam (0.1–0.5 mg/kg/h) were used for anesthesia maintenance through a continuous rate infusion (CRI) and adjusted at the discretion of a veterinarian. Animals were mechanically ventilated with a ventilator rate of 10–12 breaths/min and a tidal volume of 6–10 mL/kg with 1.4 L (70%) nitrogen and 0.6 L (30%) of oxygen (Veterinary Anesthesia Ventilator model 2002, Hallowell EMC, Pittsfield, MA). Standard monitoring of the animals was performed throughout the procedure, including monitoring of blood pressure, end-tidal carbon dioxide, heart rate, and oxygen saturation.

The carotid artery and jugular vein were exposed by blunt dissection. The carotid artery was catheterized (20 Gauge Arterial Catheterization Set FA-04018; Arrow International, Reading, PA, USA) to monitor invasive blood pressure at a frequency of either 10 Hz or 100 Hz (depending on the experimental protocol). Mean arterial pressure (MAP) was calculated as a weighted average from the arterial blood pressure (1/3 systolic + 2/3 diastolic). These data were captured using LabChart Pro software (AD Instruments, Colorado Springs, Colorado, US). The jugular vein was catheterized (6–8 French Multi-Lumen Central Venous Catheterization Set CE-12703; Arrow International) to obtain central venous access for the infusion of IV medication during surgery. Subsequently, the animal was flipped, and a dorsal laminectomy was performed, with the animal being instrumented bilaterally with pedicle screws. In 5/8 animals, a spinal cord contusion-compression injury was induced at the level of T10 as previously described^30^.

### Intraparenchymal combined OxyLite/OxyFlo probes and probe placement

**Figure 1:**
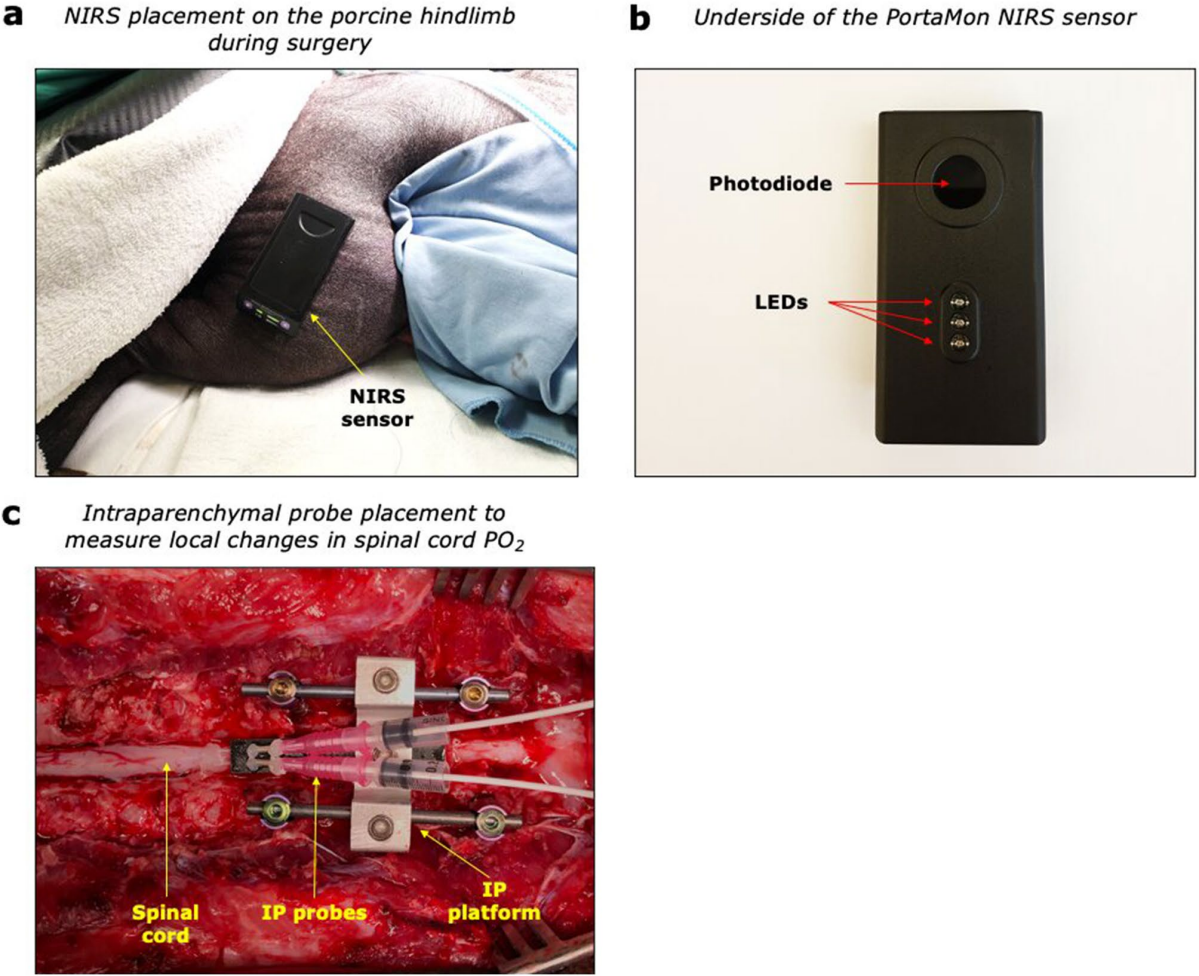
Representative experimental set-up. (**a**) Placement of NIRS sensor (PortaMon, Artinis Medical Systems, The Netherlands) on the left hindlimb muscle of a Yucatan minipig. (**b**) The underside of the NIRS sensor consists of one photodiode (light receiving element) and three LEDs (each transmitting light at two wavelengths; ~ 765 nm and ~ 850 nm). The interoptode distances from the photodiode to each LEDs are as follows: 30 mm, 35 mm and 40 mm. The three LEDs emit a total optical power of 0.88 mW, 1.78 mW and 3.67 mW, respectively. (**c**) Intra-operative placement of spinal cord intraparenchymal (IP) probes to measure local changes in spinal cord PO_2_ in response to euthanasia. IP probes were placed as part of other NIRS studies. *IP probes* intraparenchymal probes, *PO*_*2*_ partial pressure of oxygen, *NIRS* near-infrared spectroscopy.

Data on spinal cord partial pressure of oxygen (PO_2_) was collected invasively using a set of intraparenchymal sensors (NX-BF/OF/E; Oxford Optronics, Oxford, UK), which enabled us to compare changes in oxygenation measured superficially (by NIRS) to those measured from inside the spinal cord parenchyma. Each probe contains three sensors at the tip (temperature, blood flow and PO_2_; tip diameter 450 μm). The sensor measures tissue PO_2_ employing a fluorescence quenching and fiberoptic technique^33^, recorded at a sampling rate of 1 Hz. Depending on the experimental protocol, one or two intraparenchymal (IP) probes were inserted into the spinal cord parenchyma (Fig. 1c), as described in Cheung et al.^30^. The probes were inserted with the probe tips situated within the spinal cord white matter at a depth of approximately 3–4 mm under the dura. Probe placement was verified via ultrasound imaging (L14-5/38, 38 mm linear array probe, Ultrasonix RP; BK Ultrasound, Richmond, BC, Canada and VisualSonics Mx400, 30MgHz, Toronto, Canada). The probes were connected to OxyLab/OxyFlow channel monitors (Oxford Optronics, Oxford, UK), and data were streamed into LabChart Pro software (AD Instruments, Colorado Springs, Colorado, US).

### Transcutaneous near-infrared spectroscopy (NIRS) system

A transcutaneous NIRS device was used during each surgery (Fig. 1a,b). A wearable compact NIRS sensor (PortaMon/PortaLite, Artinis Medical Systems, the Netherlands) was placed on the biceps femoris muscle belly of the animal’s hindlimb. The NIRS sensor is a dual-wavelength (765 and 850 nm), continuous-wave NIRS system containing three pairs of LEDs in a spatially resolved spectroscopy (SRS) configuration and a single photodiode. Based on the SRS configuration and the modified Beer–Lambert law^27^, we obtain information on the following parameters: oxyhemoglobin (O_2_Hb, μM/L), deoxyhemoglobin (HHb, μM/L), total hemoglobin (THb = O_2_Hb + HHb, μM/L), and the hemoglobin difference (Hbdiff = O_2_Hb-HHb, μM/L)]. Furthermore, we can estimate the local tissue oxygen saturation (tissue saturation index or TSI, expressed in %) based on Eq. (1), following correction for tissue scattering. The reduced scattering coefficient was adjusted to reflect that of the human musculature (no adjustments were made to account for potential differences in human *vs* porcine tissue).

1 $$\mathrm{TSI\%}=\left(\frac{[{\mathrm{O}}_{2}\mathrm{Hb}]}{\left[{\mathrm{O}}_{2}\mathrm{Hb}\right]+[\mathrm{HHb}]}\right)\times 100\mathrm{\%}.$$

NIRS data were collected using OxySoft version 3.0.95 software (Artinis Medical Systems, the Netherlands) at a sampling frequency of 10 Hz. Each NIRS sensor was secured using double-sided adhesive tape to avoid inducing pressure on the underlying tissue (Fig. 1a). Subsequently, the NIRS sensor was covered by a sterile surgical drape for isolation from ambient light during data collection.

### Experimental protocol

The animals were euthanized with IV pentobarbital sodium administered using central jugular venous access at a standard dose of 120 mg/kg over 5 s. Pentobarbital is a short-acting barbiturate and sedative-hypnotic with a long history of use in veterinary practice for anesthesia and euthanasia^34^. The primary mode of action is causing GABA_A_-mediated CNS dysfunction, critically depressing medullary centres in the brainstem, leading to rapid onset apnea^34,35^, and cardiovascular collapse with features of bradycardia^36,37^**,** refractory hypotension^35,37^ and reduced myocardial contractility^37^. The start time of pentobarbital administration was marked in LabChart. During pentobarbital administration, pigs continued to receive mechanical ventilation and oxygenation. Thus, the mechanism of cardiac arrest was not secondary to the pentobarbital-induced apnea (i.e., respiratory arrest, which can lead to secondary cardiac arrest) but rather primary cardiac collapse. Upon cessation of a cardiac rhythm on transesophageal ECG, ventilation was discontinued, and death was confirmed by a registered veterinary technician upon auscultation for breathing and heart sounds and checking for a palpable peripheral pulse (ear or tail). During this time, all monitoring remained switched on, and NIRS, invasive blood pressure, and spinal cord PO_2_ signals continued to be captured for up to 5 min post-euthanasia.

### Signal processing and data analysis

#### Transcutaneous NIRS versus systemic MAP and spinal cord PO_2_ during euthanasia

All data were resampled at 10 Hz for subsequent analysis. In all animals, NIRS and LabChart data were aligned offline by the presence of artifact patterns in the signals. Such patterns result from various motion artifacts and can be used to identify common events in the time series measurements. Data were assessed for normal distribution using a D’Agostino Pearson omnibus normality test. The time of euthanasia was identified from an event marker inserted into LabChart during surgery. The analysis window was identified as 10 min before the event marker was inserted and then 5 min after the event had been added (15 min total). Relative NIRS parameters (O_2_Hb, HHb, THb and Hbdiff) were z-score normalized to the first 5 min of this window and are expressed in standard deviation units (s.d.u.). We examined mean arterial pressure (MAP), NIRS TSI (%), and spinal cord PO_2_ values in the last 10 s of the recording after euthanasia and compared them to average pre-euthanasia baseline values (collected over 60 s) using a paired Student’s t-test (level of significance p < 0.05).

#### Time to first detectable change in NIRS TSI (%), NIRS O_2_Hb, spinal cord PO_2_ and MAP signals in response to pentobarbital infusion

Comparisons between the time of pentobarbital infusion to the first detectable change in NIRS TSI (%), spinal cord PO_2_ vs invasive MAP signals were calculated using a paired Student’s t-test. We defined the “first detectable change” in the descending signal as the time from euthanasia marker insertion in LabChart to the first drop of the signal two standard deviations below its baseline (the baseline value was defined as the mean value during the last-minute pre-pentobarbital infusion; see Fig. 4a).

#### Transcutaneous NIRS versus arterial blood pressure-derived pulse rate and pulse amplitude changes in response to euthanasia

To obtain pulse rate and amplitude from carotid arterial blood pressure (ABP) catheter measurements and NIRS O_2_Hb signal dynamics (selected due to prominent cardiac components in their respective time series), short-time Fourier transform (STFT) analyses (fs = 10 Hz; FFT length of 528 samples; window overlap of 24%) were carried out on z-score normalized signals. Three separate 60-s analysis windows were selected. These represent (1) baseline signals, (2) the 60-s interval immediately following pentobarbital infusion and (3) post-euthanasia signals. Pulse rates (Hz) and pulse amplitudes (dB) were derived from these STFT windows (displayed as mean ± 95% confidence intervals). Group differences (ABP vs O_2_HB-derived pulse amplitude and pulse rate) were analyzed using a two-tailed, unpaired Student’s *t*-test or a repeated measure ANOVA (level of significance *p* < 0.05) for multiple group comparisons.

All analysis was carried out using GraphPad Prism 9.0.0 software (GraphPad Software, La Jolla, CA, USA), MATLAB (version R2021a, Natick, Massachusetts, USA) or Python libraries (Python Software Foundation). Data are presented as mean ± standard deviation (SD) (or ± standard error of the mean (SEM) when otherwise specified).

### Institutional review board statement

All animal protocols and procedures used for this study were in accordance with the policies of the Canadian Council on Animal Care and were reviewed and approved by the Institutional Animal Care and Use Committee (IACUC) of the University of British Columbia (protocol No. A20-0217 and A16-0311, approved Oct 26, 2020, and Feb 17, 2017, respectively), and the Animal Care and Use Review Office (ACURO) of the U.S. Army and the Naval Information Warfare Centre [Award No. N6600120-2-4046, approved Dec-21-2020 (NIWC, USAMRMC Protocol No. NIWC-7783.e002), and Award No. W81XWH-16-1-0602, approved May-16-2017, respectively].

## Results

### Transcutaneous NIRS versus systemic MAP and spinal cord PO_2_ during euthanasia

**Figure 2:**
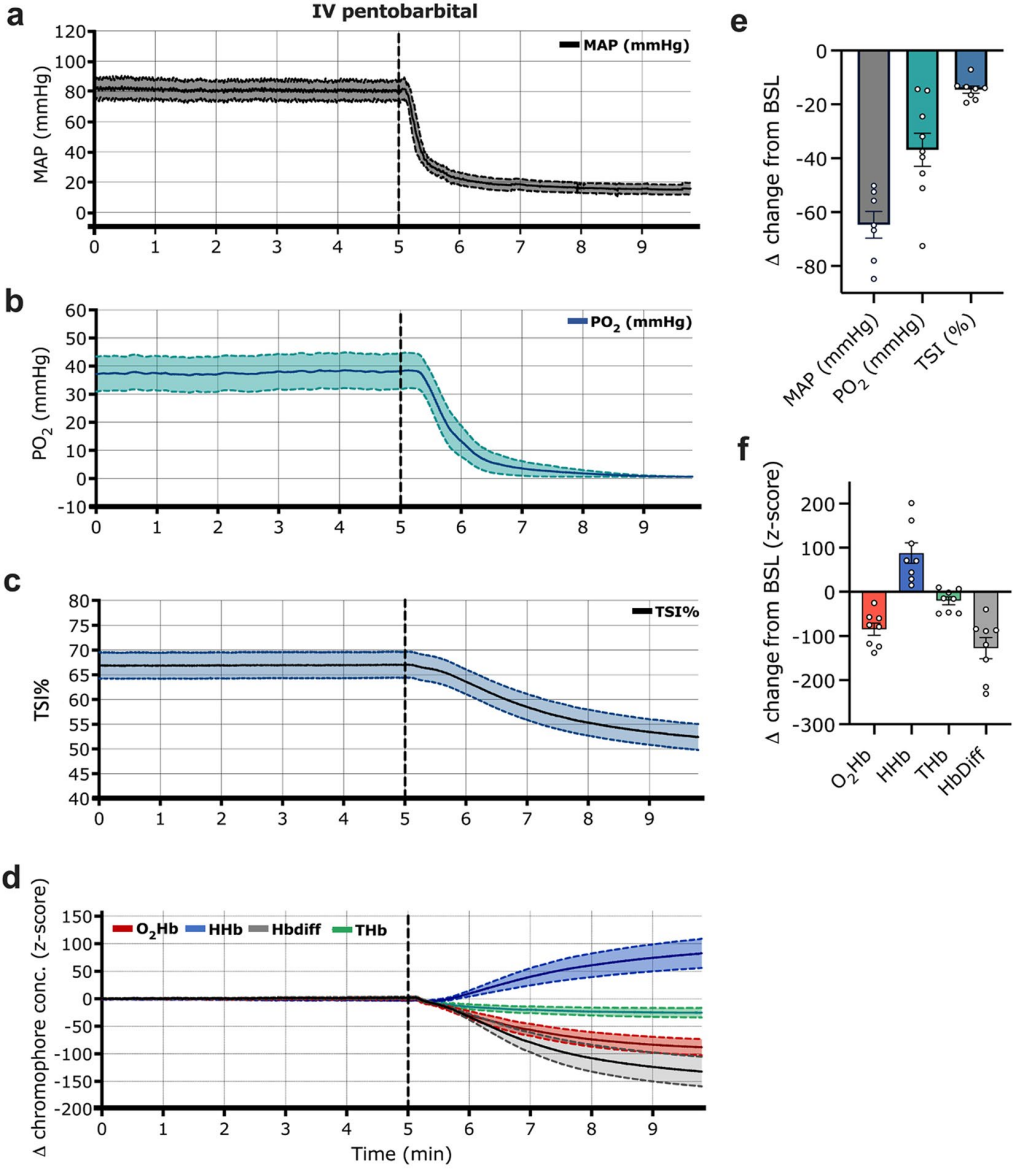
Transcutaneous NIRS, carotid blood pressure and spinal cord PO_2_ responses to pentobarbital administration. Averaged traces from (**a**) MAP (n = 7 animals, ± SEM), (**b**) spinal cord PO_2,_ (n = 6, ± SEM), (**c**) TSI (%) (n = 8, ± SEM), and (**d**) relative changes in NIRS parameters of O_2_Hb, HHb, THb and Hbdiff (n = 8 ± SEM). The 5-min timepoint (dashed line) denotes the time of pentobarbital administration. (**e**,**f**) Delta changes to euthanasia relative to a 60-s pre-euthanasia baseline. *TSI (%)* tissue saturation index, *Hbdiff* hemoglobin difference, *O*_*2*_*Hb* oxygenated hemoglobin, *HHb* deoxygenated hemoglobin, *THb* total hemoglobin, *MAP* mean arterial pressure, *PO*_*2*_ partial pressure of oxygen, *BSL* baseline, *EUT* euthanasia.

**Figure 3:**
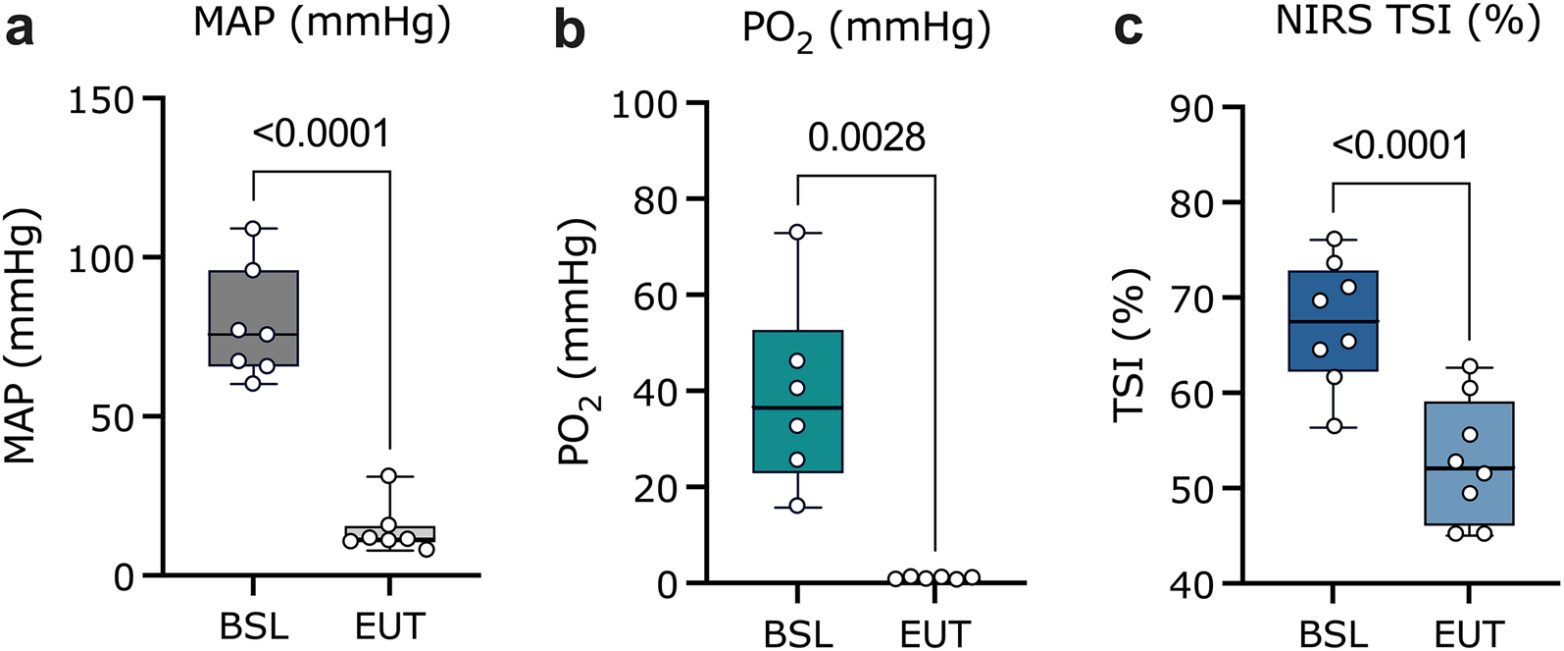
Pre- and post-euthanasia comparison of carotid MAP, spinal cord PO_2_ and TSI (%) measures. Box plots (IQR + min and max values) of pre-euthanasia (BSL; averaged over 60 s pre-pentobarbital infusion) and post-euthanasia values (EUT; averaged over the last 10 s of the recording) in (**a**) MAP (mmHg) (n = 7, *p* < 0.0001) (**b**) spinal cord PO_2_ (mmHg) (n = 6, *p* = 0.0028, and (**c**) TSI (%) (n = 8, *p* < 0.0001). Group comparisons were analyzed using a paired student’s t-test (level of significance *p* < 0.05). *TSI (%)* tissue saturation index, *MAP* mean arterial pressure, *PO*_*2*_ partial pressure of oxygen, *BSL* baseline, *EUT* euthanasia.

Compared to baseline, carotid MAP decreased by 64.68 mmHg ± 13.08 in the 5 min post-pentobarbital administration (n = 7; Student’s t-test, *p* < 0.0001; Table 1, Figs. 2a, 3a). The mean decrease in spinal cord PO_2_ was 38.16 mmHg ± 20.04 (n = 6; Student’s t-test, *p* = 0.0028; Table 1, Figs. 2b, 3b). Similarly, we observed a statistically significant drop in the TSI (%) (n = 8; Student’s t-test, *p* < 0.0001; Table 1, Figs. 2c,e, 3c), which dropped on average by 14.50% ± 3.80 within the first 5 min. Relative NIRS measures showed (Fig. 2d,f): a decrease in oxygenated hemoglobin (O_2_Hb; − 84.33 s.d.u. ± 38.65); a concomitant increase in the deoxygenated hemoglobin portion (HHb; 87.66 s.d.u. ± 65.62); and a decrease in the parameter of the hemoglobin difference (Hbdiff; − 127.23 s.d.u. ± 67.75) in all animals.

**Table 1:** Carotid blood pressure, spinal cord PO_2_ and transcutaneous NIRS measurements at pre-euthanasia baseline (averaged over 60 s), 5-min post euthanasia (average of the last 10 s of recording) and ΔMAP, spinal cord PO_2_ and TSI (%) values. (Note on missing values: MAP values from one experiment were excluded as signal quality significantly deteriorated shortly after pentobarbital administration. In 2/8 experiments, data on spinal cord PO_2_ was unavailable due to modifications in the original experimental protocol). *TSI (%)* tissue saturation index, *MAP* mean arterial pressure, *PO*_*2*_ partial pressure of oxygen.

Animal #	Carotid MAP (mmHg)			Spinal cord PO_2_ (mmHg)			Hindlimb TSI (%)		
	Baseline	Euthanasia	Δ	Baseline	Euthanasia	Δ	Baseline	Euthanasia	Δ
1	109.08	31.02	− 78.06	15.74	0.87	− 14.87	56.35	49.27	− 7.08
2	67.32	11.55	− 55.77	25.26	0.79	− 24.47	61.52	45.00	− 16.52
3	75.69	10.86	− 64.83	32.40	0.48	− 31.91	65.26	51.36	− 13.90
4	65.71	15.53	− 50.18	72.91	0.38	− 72.53	70.96	52.61	− 18.35
5	95.94	11.15	− 84.79	45.96	0.32	− 45.65	73.50	60.34	− 13.16
6	60.21	7.76	− 52.45	NA	NA	NA	69.55	55.43	− 14.13
7	77.09	10.38	− 66.71	NA	NA	NA	64.40	45.01	− 19.39
8	NA	NA	NA	40.26	0.70	− 39.56	76.08	62.63	− 13.45
*Mean*	78.72	14.04	− 64.68	38.75	0.59	− 38.16	67.20	52.71	− 14.50
*SD*	17.66	7.83	13.08	19.87	0.23	20.04	6.54	6.51	3.80

### Time to first change in NIRS TSI (%), NIRS O_2_Hb, spinal cord PO_2_ and MAP signals in response to pentobarbital infusion

**Figure 4:**
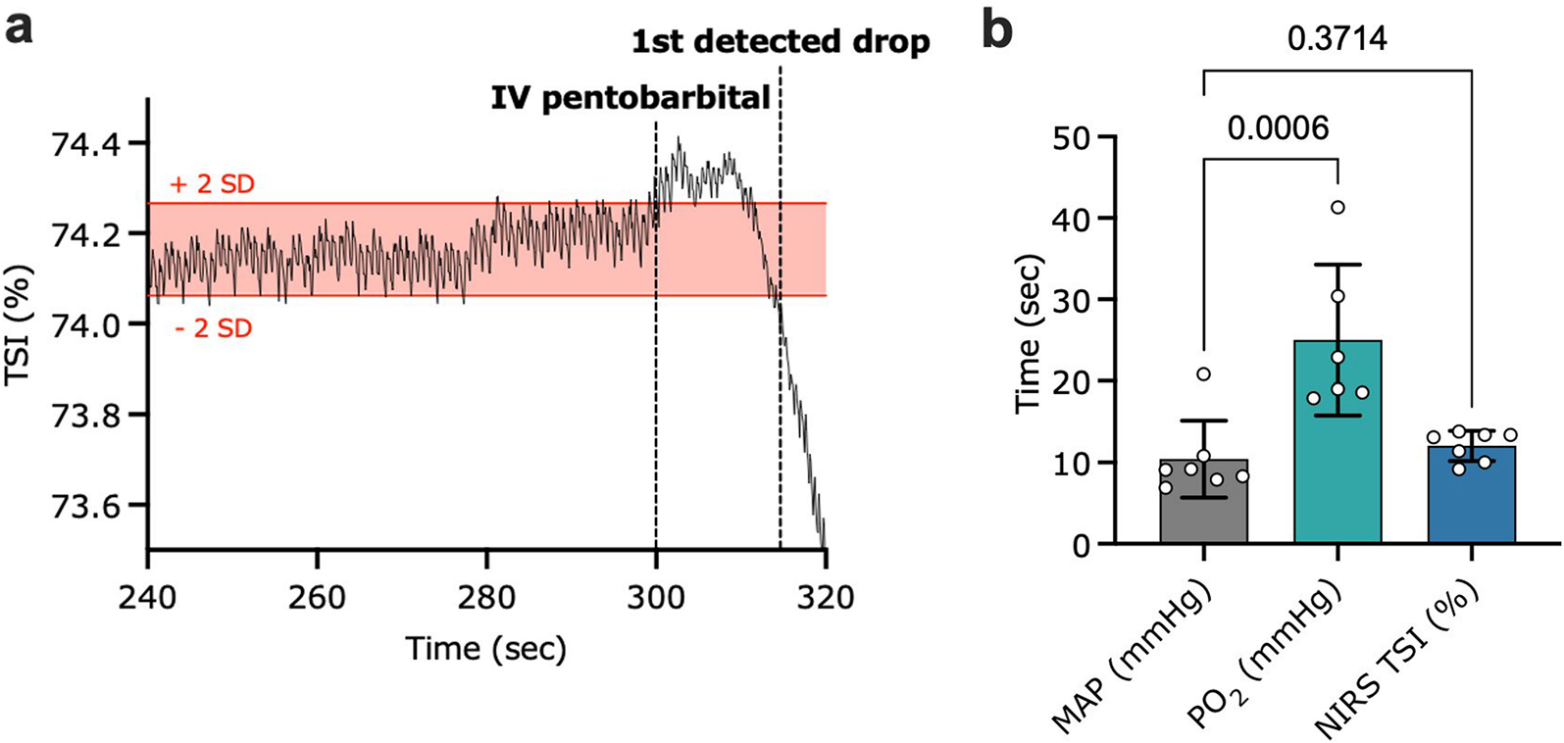
Euthanasia-to-first change in hemodynamics for MAP, spinal cord PO_2_ and NIRS-derived TSI (%) and O_2_Hb. (**a**) Representative figure of a NIRS TSI (%) trace pre- and immediately following pentobarbital infusion. The first detected drop in the signal was defined as the first value two standard deviations below baseline (established over the last 60 s prior to pentobarbital administration). (**b**) Time (sec) to the first change in carotid MAP, spinal cord PO_2_ and hindlimb TSI (%) signals in response to euthanasia ± SD. MAP vs TSI (%) (n = 7; *p* = 0.3714), and MAP vs spinal cord PO_2_ (n = 6, *p* = 0.0006) were analyzed using paired student’s t-tests (level of significance* p* < 0.05); *TSI (%)* tissue saturation index, *MAP* mean arterial pressure, *PO*_*2*_ partial pressure of oxygen.

The time to first change in MAP was observed at 10.43 ± 4.73 s (Fig. 4b). NIRS-derived TSI (%) dropped at 12.04 ± 1.85 s (Fig. 4b). In 5/6 animals (as information on spinal cord PO_2_ was unavailable in 2/8 animals), spinal cord PO_2_ measures were the slowest to respond, taking 25.02 ± 9.237 s to register a change in spinal cord PO_2_ two standard deviations below the baseline (Fig. 4b). The difference in detection time between MAP and NIRS-derived TSI (%) (n = 7, paired Student’s t-test, *p* = 0.3714, level of significance *p* < 0.05) was not significant.

### Transcutaneous NIRS versus arterial blood pressure-derived pulse rate and pulse amplitude changes in response to euthanasia

**Figure 5:**
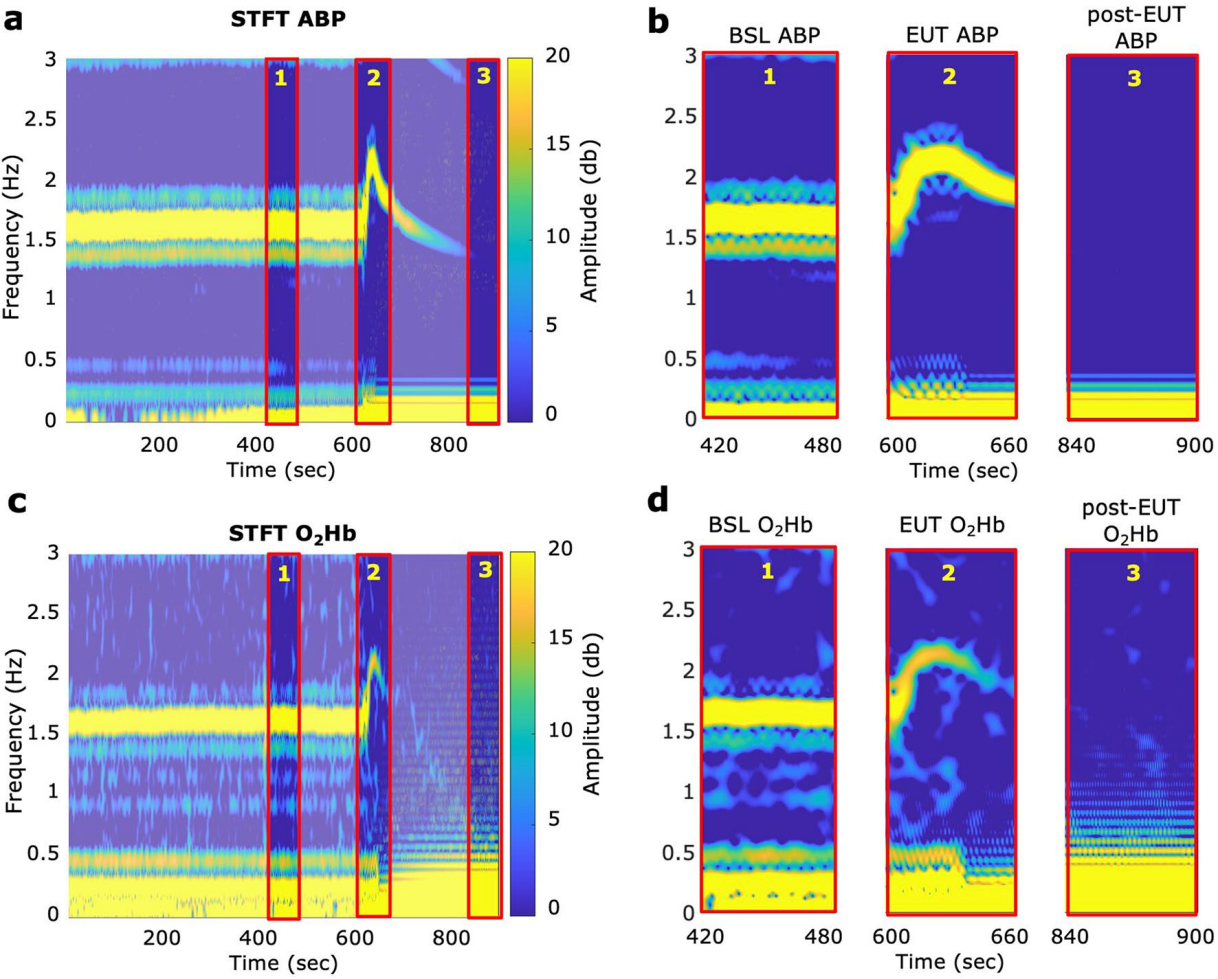
Representative short-time Fourier transform analyses. STFT colormaps of (**a**) carotid ABP and (**c**) NIRS O_2_Hb signals over a 15-min window. Pentobarbital infusion commenced at 600 s. (**b**,**d**) Three analysis windows were selected for subsequent analysis, denoting different stages of euthanasia. *O*_*2*_*Hb* oxygenated hemoglobin, *ABP* arterial blood pressure, *BSL* baseline, *EUT* euthanasia, *STFT* short time Fourier transform (for STFT colourmaps from individual animals, see Supplementary Figs. 1–8).

**Figure 6:**
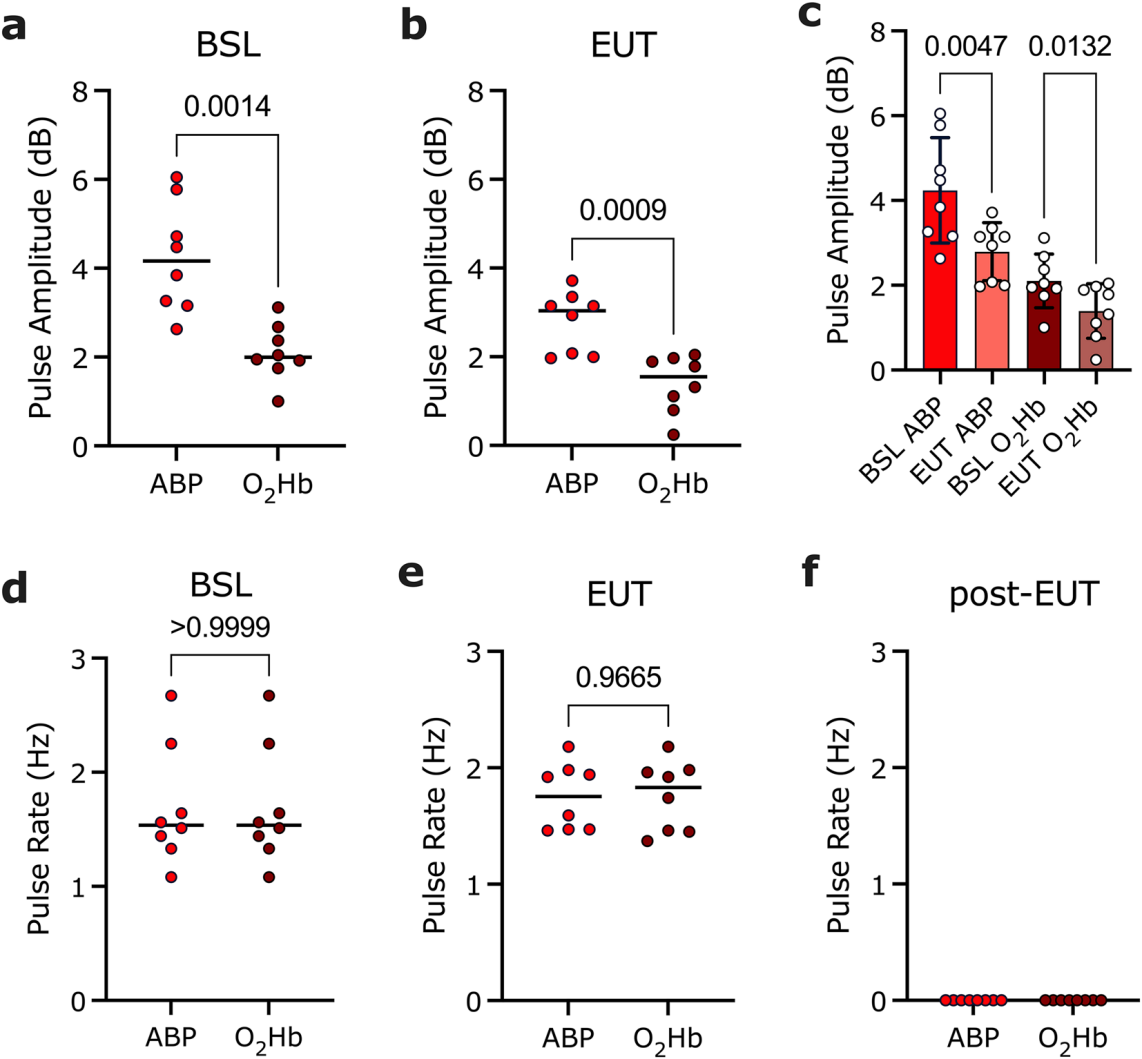
Comparison of ABP and NIRS-derived pulse rate and amplitude changes in response to pentobarbital administration. Mean ABP and O_2_Hb-derived pulse amplitudes (dB) (n = 8) during (**a**) baseline (*p* = 0.0014) and (**b**) euthanasia (*p* = 0.0009) (Student’s *t*-test, level of significance *p* < 0.05). (**c**) ABP and O_2_Hb pulse amplitudes (dB) at BSL vs EUT (Repeated measure ANOVA, adjusted p-values: ABP BSL vs EUT *p* = 0.0047 and O_2_Hb BSL vs EUT *p* = 0.0132; level of significance *p* < 0.05). Mean ABP and O_2_Hb-derived pulse rates (Hz) (n = 8) during (**d**) baseline (*p* > 0.99), (**e**) euthanasia (*p* = 0.97) and (**f**) post-euthanasia (unable to calculate *p*). Group comparisons were analyzed using a student’s *t*-test (level of significance *p* < 0.05). *ns* not significant, *O*_*2*_*Hb* oxygenated hemoglobin, *ABP* arterial blood pressure, *BSL* baseline, *EUT* euthanasia.

In all animals, O_2_Hb and ABP-calculated pulse rates were comparable (see Fig. 5 for representative STFT analysis), and no statistically significant difference was detected between O_2_Hb and ABP-derived pulse rates within pre-euthanasia (*p* > 0.99), euthanasia (*p* = 0.97), and post-euthanasia analysis windows (n = 8, Student’s *t*-test, level of significance *p* < 0.05; Table 2, Fig. 6d–f). In 6/8 animals, we noted a transient increase in pulse rate upon pentobarbital infusion (see Supplementary Fig. S1–S3, S5–7). In 2/8 animals, the pulse rate started to decline immediately after euthanasia induction (see Supplementary Figs. S4 and S8).

**Table 2:** Summary statistics of NIRS-derived O_2_Hb versus ABP-derived pulse rates (Hz) ± 95% confidence intervals for three 60-s intervals (pre-euthanasia, immediately following pentobarbital infusion and post-euthanasia). *ABP* arterial blood pressure, *O*_*2*_*Hb* oxygenated hemoglobin.

Pulse rate (Hz) ± 95% CI									
		Animal #1	Animal #2	Animal #3	Animal #4	Animal #5	Animal #6	Animal #7	Animal #8
Pre-euthanasia	ABP	1.44 ± 0.14	1.51 ± 0.02	1.56 ± 0.14	2.67 ± 0.01	1.33 ± 0.02	1.08 ± 0.02	1.64 ± 0.02	2.25 ± 0.00
	NIRS O_2_Hb	1.44 ± 0.02	1.51 ± 0.03	1.56 ± 0.14	2.67 ± 0.01	1.33 ± 0.03	1.08 ± 0.02	1.64 ± 0.02	2.25 ± 0.01
During euthanasia	ABP	1.47 ± 0.21	1.47 ± 0.15	1.46 ± 0.54	2.18 ± 0.39	1.94 ± 0.49	1.92 ± 0.39	1.98 ± 0.26	1.59 ± 0.77
	NIRS O_2_Hb	1.45 ± 0.19	1.46 ± 0.14	1.37 ± 0.44	2.18 ± 0.38	1.98 ± 0.38	1.92 ± 0.44	1.96 ± 0.28	1.74 ± 0.54
Post-euthanasia	ABP	0 ± 0	0 ± 0	0 ± 0	0 ± 0	0 ± 0	0 ± 0	0 ± 0	0 ± 0
	NIRS O_2_Hb	0 ± 0	0 ± 0	0 ± 0	0 ± 0	0 ± 0	0 ± 0	0 ± 0	0 ± 0

Pulse amplitudes (dB) were stronger in ABP compared to NIRS O_2_Hb signals (Fig. 6a,b). This difference was significant during baseline (n = 8, Student’s t-test, *p* = 0.0014, level of significance *p* < 0.05; Fig. 6a) and following pentobarbital administration (n = 8, Student’s t-test, *p* = 0.0009, level of significance *p* < 0.05; Fig. 6b). Lastly, we saw statistically significant decreases within ABP and NIRS-derived O_2_Hb pulse amplitudes in response to pentobarbital administration compared to baseline (n = 8, repeated measure ANOVA, adjusted p-values: ABP BSL *vs* EUT *p* = 0.0047 and O_2_Hb BSL *vs* EUT *p* = 0.0132; level of significance *p* < 0.05; Fig. 6c).

## Discussion

Using a porcine model of primary cardiac arrest, we examined changes in NIRS measurements during cardiac arrest and compared them to invasive measurements of spinal cord oxygenation and blood pressure monitoring. While there have been several reports using cerebral NIRS monitoring to assess return to spontaneous circulation (ROSC) and quality of resuscitation post-SCA^38–45^, the use of transcutaneous NIRS monitoring in the setting of cardiac arrest detection has not been explored and presents a potentially novel field of research. Our results demonstrated a quick and clear change in NIRS signals during and after cardiac arrest, similar to invasive hemodynamic measurements. Overall, these findings demonstrate the utility of muscle-NIRS-based monitoring for SCA detection; however, further studies are needed to establish its efficacy.

In this study, we used pentobarbital sodium to induce euthanasia in our porcine model. Pentobarbital depresses medullary centres in the brainstem, causing rapid death by respiratory depression^34,35^ and cardiovascular collapse^35–37^. There are two main models of cardiac arrest: (1) primary cardiac arrest with a direct insult to cardiac tissue, causing cessation of effective cardiac output and resultant whole-body hypoxia; and (2) primary respiratory arrest, causing whole-body hypoxia (including that of cardiac tissue), resulting in secondary cardiac arrest. Although phenobarbital causes respiratory depression, in this study, mechanical ventilation and oxygenation were continued after pentobarbital administration (preventing hypoxia), consistent with primary cardiac arrest. Further research is required to investigate physiological changes with respiratory arrest leading to secondary cardiac arrest, as is more frequently encountered in infants, children, and adults with opioid-related cardiac arrest or other respiratory etiologies^46^. Since NIRS measures tissue oxygenation, it may have high utility in detecting respiratory arrest before cardiac arrest.

We observed considerable inter-animal variability in pre-euthanasia measures of spinal cord PO_2_, which may reflect variations in the IP probes’ placement and small sampling area (reported to be approximately 0.5–1 mm^3,47^). These limitations result in spinal cord PO_2_ readings that may be highly sensitive to differences in tissue composition and local microvasculature. Similarly, baseline MAP measures displayed notable variability, which is not surprising given the wide range of physiological blood pressures. TSI (%) pre-euthanasia was comparable across animals, indicating that regional oxygen saturation is preferentially maintained within a specific range in the animal’s hindlimb in the anesthetized animal.

While MAP and spinal cord PO_2_ dropped to levels below those observed for living tissue within the first 5 min post-euthanasia induction, we observed a smaller TSI (%) decrease (14.50% ± 3.80). In one instance, TSI (%) dropped from 76.08% to 62.63% (animal #8, Table 1). Due to a comparatively high TSI (%) at baseline, this decrease associated with complete cessation of cardiac function in this animal could be within the physiological TSI (%) range of another animal (see animals #1–2, Table 1). This suggests that a particular TSI (%) value may not be sufficient to detect sudden cardiac events but instead gains diagnostic value if interpreted in the context of NIRS-derived hemodynamic trends, pulse rate changes, and patient-specific baseline TSI (%) measures. Importantly, TSI (%) does not reflect arterial oxygen saturation (i.e. the amount of oxygen in the arterial stream available to tissues and organs), but rather is an index of oxygenated to total hemoglobin at a microvascular tissue level^48^ composed of both arterial and venous components and thus most likely includes “inaccessible” oxygen trapped within venous compartments. Furthermore, it is crucial to consider that other local and systemic conditions could lead to similar decreases in the TSI (%). This should be emphasized when placed in the broader context of discussing the specificity of using NIRS-derived changes in the setting of SCA detection. Our group has recently shown in our porcine model that controlled periods of hypoxia to a SpO_2_ target of 70% lead to a TSI% decrease of 10.5 ± 1% as recorded by the PortaMon NIRS sensor from the animal’s hindlimb^49^. While clinically, such severe episodes of hypoxia are unlikely to be encountered on a routine basis; other investigators have reported decreases in NIRS-derived tissue saturation indices in various clinical settings that lead to tissue hypoperfusion, including vascular disease^50^ and shock^51–53^.

We further observed that the rate of change for both MAP and spinal cord PO_2_ was highest within the first minute post-pentobarbital infusion once a significant decrease in the signal below baseline was detected. This is consistent with previous reports from a canine study where pentobarbital caused MAP to decrease to undetectable levels at a mean time of 52.6 s^54^. The rate of TSI (%) change was slower and showed a slightly decreasing trend at 5 min post-euthanasia. MAP reflects the mean blood pressure over a single cardiac cycle and is thus highly susceptible to the loss of effective ventricular contractions and decreased cardiac output. On the other hand, NIRS measures the “effect” of this loss of contractile activity on the underlying tissue, which at the time of cardiac arrest is still metabolically active^55,56^. Without systemic circulation, this continued oxygen exchange may underlie the steady decline in NIRS-derived muscle oxygen saturation over a prolonged period.

Notably, the comparison between the time from pentobarbital administration to the first detectable drop in NIRS-derived TSI (%) from a pre-defined baseline demonstrates the high sensitivity of NIRS to detect systemic changes in tissue oxygenation non-invasively. In contrast, the first detectable drop in spinal PO_2_ occurred significantly slower. Spinal cord PO_2_ represents the local partial pressure of oxygen (i.e., “dissolved” oxygen) in the tissue and thus provides a readout of oxygen availability at a cellular level. As oxygen is initially released from hemoglobin binding sites to meet the metabolic demand of the tissue, local spinal cord PO_2_ will remain stable until the metabolic demand of the tissue surpasses the oxygen-releasing capacity of hemoglobin. However, other factors should also be considered, such as potential delays in IP probe response times and low sampling rates.

We also compared mean NIRS O_2_Hb- and carotid ABP-derived pulse amplitudes at three different time points (pre-, during- and post-euthanasia). In all animals, ABP-derived pulse amplitudes were stronger than those derived from NIRS, both before and immediately following intravenous pentobarbital infusion. This is unsurprising as both signals are derived from different sources. Invasive blood pressure monitoring is achieved by monitoring cyclical pressure changes from one of the major vessels bifurcating from the aorta. NIRS, on the other hand, measures pulse changes indirectly by capturing the effect of pulsatile blood flow on underlying tissue dynamics at a site distal to the heart. As previously discussed, NIRS will capture changes occurring on a microvascular level, capturing contributions from arteriolar, capillary, and venular blood alike. Importantly, venules and capillaries do not contribute significantly to pressure waveform propagation while still making up a significant portion of the NIRS signal. This will contribute to the reduced pulse amplitude observed in the NIRS signal compared to the ABP. Of note is that in six out of eight animals, we observed the presence of cardiac activity after death had been clinically confirmed, consistent with previous reports that such signals may persist for 5.5–16 min after pentobarbital infusion^54^. Notably, both ABP- and NIRS-derived mean pulse amplitudes decreased during euthanasia (compared to baseline), indicating a decrease in cardiac inotropic activity captured by both systems. Real-time NIRS-based pulse amplitude monitoring presents a potentially new avenue of how cardiac arrest may be identified, encouraging further investigation.

We also observed a strong agreement between NIRS and carotid ABP-derived pulse rates during the three distinct analysis windows, as revealed by short-time Fourier transform analyses. Notably, in six out of eight animals, we observed transient increases in heart rate during and immediately following the infusion of pentobarbital, consistent with previous findings^54^. This suggests that, while overall NIRS-derived pulse amplitudes are weaker at this more distal body site, the NIRS system captures changes in pentobarbital-induced pulse rate pre-and during cardiac dysfunction at a high sensitivity when assessed against invasive blood pressure monitoring. Based on these results, we conclude that transcutaneous NIRS may be used in the setting to provide similar information. We could not perform this assessment on IP-derived parameters, as the low sampling frequency of the combined OxyLite/OxyFlow system prevented us from detecting pulsatile blood flow changes occurring on a beat-to-beat basis.

Several other technologies have been examined for non-invasively detecting out-of-hospital SCA. These include using wearable ECG-based monitoring/defibrillation systems that have shown promise in reducing mortality in high-risk patients when worn following myocardial infarction^57^ and left ventricular dysfunction^58^. Furthermore, photoplethysmography (PPG) smartwatch/bracelet-based heart rate monitoring systems are available^59–63^. However, as PPG sensors rely on pulsatile blood flow, they become less reliable or may be unable to return a measurement during periods of systemic hypotension, hypothermia or poor peripheral circulation^24,64,65^. As such, PPG monitoring has been predominately investigated for its ability to capture changes in heart rate and heart rate variability as a way to monitor return to spontaneous circulation (ROSC) during cardiopulmonary resuscitation^59,66^ and the long-term monitoring and screening of cardiac arrhythmia as previously reviewed^62^. The ability of NIRS to capture the cessation of cardiac pulsations while monitoring tissue oxygenation changes (even in the absence of a pulse waveform) suggests that NIRS could be a helpful tool to detect cardiac arrest in an out-of-hospital setting and track its progression until ROSC is resumed. This would further facilitate investigation into a potential relationship between NIRS-derived oxygenation changes during cardiac arrest progression and clinical prognosis. Lastly, smart devices have been used to capture agonal breathing patterns to detect SCA^67^. Agonal breathing is a brainstem reflex resulting from severe hypoxia. However, agonal breathing only occurs in 55% of witnessed cardiac arrest cases^68^. The resulting low sensitivity constraints widespread use of such monitoring in the setting of SCA detection.

Overall, our results suggest that NIRS may have utility for the rapid detection of cardiac arrest. Recent technical advancements in electro-optic components and NIRS technology enable the development of compact, wearable, low-power, sensitive, cheap, and flexible NIRS sensors. Integrating miniaturized NIRS sensors with other relevant wearable biosensors may form novel multi-modal sensing approaches for extensive monitoring of vital signs and prediction and rapid diagnosis of critical cardiac dysfunctions.

There are several limitations to what we report. First, while there are many different etiologies and physiological variations within human SCA, our pentobarbital-induced porcine model of euthanasia SCA may differ from the human experience. This is particularly relevant, as the time from pentobarbital infusion to confirmed death is unknown, which limits our ability to extrapolate findings to a more clinically relevant scenario. Secondly, since physiological data were captured with different recording software and were subsequently aligned offline by the presence of artifact patterns in the signal, they are susceptible to minor errors in alignment. However, given the unique nature of artifact patterns in the signals, we expect minimal misalignment within data sets, if any. Similarly, we expect some inter-animal variability from discrepancies in the exact timing of euthanasia marker insertion denoting IV pentobarbital administration. Lastly, it is essential to note that no adjustments to scattering coefficients were made to account for potential human *vs* porcine differences within the calculation of the tissue saturation index, which is established for human use. Without making model adjustments, we postulate that the following inter-species differences could affect the calculation and should be further explored in subsequent studies: (1) skin pigmentation, (2) skin texture, (3) skin thickness and (4) subcutaneous adipose tissue thickness.

## Conclusions

Transcutaneous NIRS monitoring may present a novel, feasible, non-invasive SCA detection approach that warrants further investigation. The ability of transcutaneous NIRS to simultaneously capture the cessation of cardiac pulsatile activity while monitoring changes in tissue oxygenation may result in the development of novel, wearable optical sensors for the early detection of SCA and its subsequent tracking. This may improve the management of unwitnessed SCAs and accelerate access to life-saving interventions.

## Supplementary Information

Supplementary Figures.

## Data Availability

The datasets generated during and/or analyzed during the current study are available from the corresponding author upon reasonable request.
