# Supplementary material for: Evaluation of transcutaneous near-infrared spectroscopy for early detection of cardiac arrest in an animal model
Source: Sci Rep. 2023 Mar 20;13:4537. doi: 10.1038/s41598-023-31637-1 (PMC10027843; doi:10.1038/s41598-023-31637-1)
Supplement: Supplementary file 1 — Supplementary Figures. [file 41598_2023_31637_MOESM1_ESM.docx]

**Evaluation of transcutaneous near-infrared spectroscopy for early detection of cardiac arrest in an animal model**

**Katharina Raschdorf^1,2^, Arman Mohseni^1^, Kaavya Hogle^3^, Amanda Cheung^1^, Kitty So^1^, Neda Manouchehri^1^, Mahsa Khalili^4^, Saud Lingawi^3^, Brian Grunau^4^, Calvin Kuo^3^, Jim Christenson^4^, Babak Shadgan^1,2,3,5^***

1 International Collaboration on Repair Discoveries, 818 West 10th Avenue, Vancouver, BC V5Z 1M9, Canada

2 Department of Neuroscience, University of British Columbia, 2215 Wesbrook Mall, Vancouver, BC V6T 1Z3, Canada

3 School of Biomedical Engineering (SBME), University of British Columbia, 2222 Health Sciences Mall, Vancouver, BC V6T 1Z4, Canada

^4^ Department of Emergency Medicine, University of British Columbia and St. Paul’s Hospital, Vancouver, B.C., Canada

^5^ Department of Orthopaedics, University of British Columbia, 2775 Laurel Street, Vancouver, BC V5Z 1M9, Canada

**Supplementary material**

**STFT analysis (animal #1)**

**
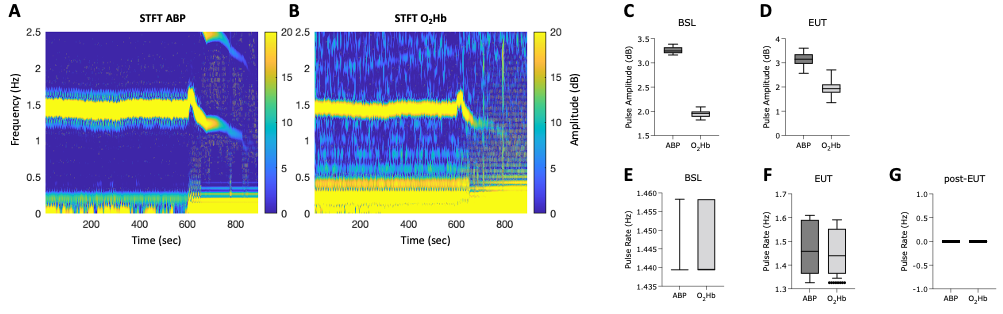
**

**Supplementary Figure S1** STFT analysis (animal #1) of **(A)** carotid ABP and (**B**) NIRS O_2_Hb signals over a 15-minute window. (**C-D**) Differences in the mean and variance of ABP and O_2_Hb-derived pulse amplitudes (dB) and (**E-G**) pulse rates (Hz) at baseline, during- and post-euthanasia. O_2_Hb -oxygenated hemoglobin; ABP – arterial blood pressure; BSL – baseline; EUT – euthanasia.

**STFT analysis (animal #2)**


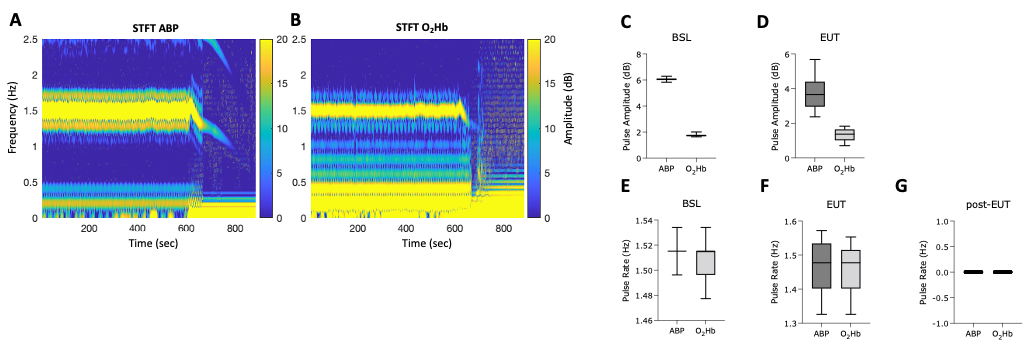


**Supplementary Figure S2** STFT analysis (animal #2) of **(A)** carotid ABP and (**B**) NIRS O_2_Hb signals over a 15-minute window. (**C-D**) Differences in the mean and variance of ABP and O_2_Hb-derived pulse amplitudes (dB) and (**E-G**) pulse rates (Hz) at baseline, during- and post-euthanasia. O_2_Hb -oxygenated hemoglobin; ABP – arterial blood pressure; BSL – baseline; EUT – euthanasia.

**STFT analysis (animal #3)**

**
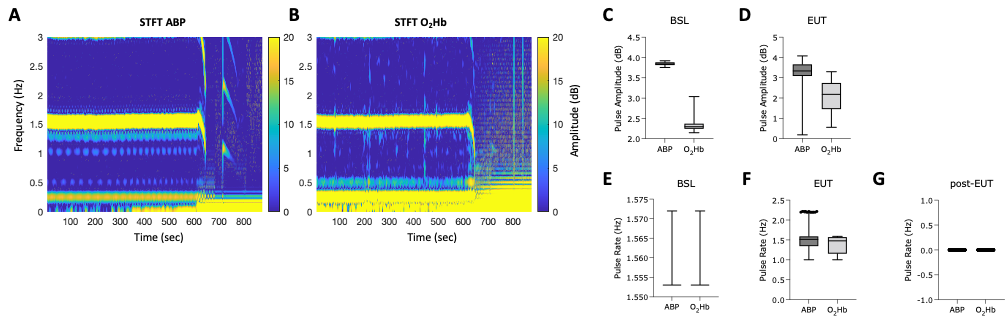
**

**Supplementary Figure S3** STFT analysis (animal #3) of **(A)** carotid ABP and (**B**) NIRS O_2_Hb signals over a 15-minute window. (**C-D**) Differences in the mean and variance of ABP and O_2_Hb-derived pulse amplitudes (dB) and (**E-G**) pulse rates (Hz) at baseline, during- and post-euthanasia. O_2_Hb -oxygenated hemoglobin; ABP – arterial blood pressure; BSL – baseline; EUT – euthanasia.

**STFT analysis (animal #4)**


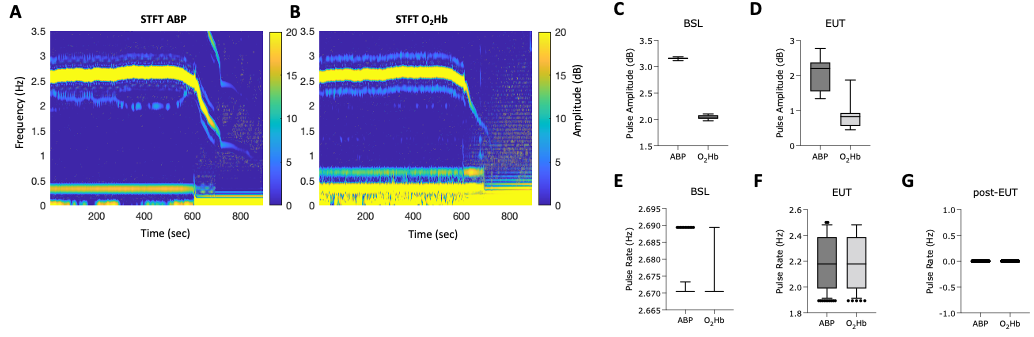


**Supplementary Figure S4** STFT analysis (animal #4) of **(A)** carotid ABP and (**B**) NIRS O_2_Hb signals over a 15-minute window. (**C-D**) Differences in the mean and variance of ABP and O_2_Hb-derived pulse amplitudes (dB) and (**E-G**) pulse rates (Hz) at baseline, during- and post-euthanasia. O_2_Hb -oxygenated hemoglobin; ABP – arterial blood pressure; BSL – baseline; EUT – euthanasia.

**STFT analysis (animal #5)**


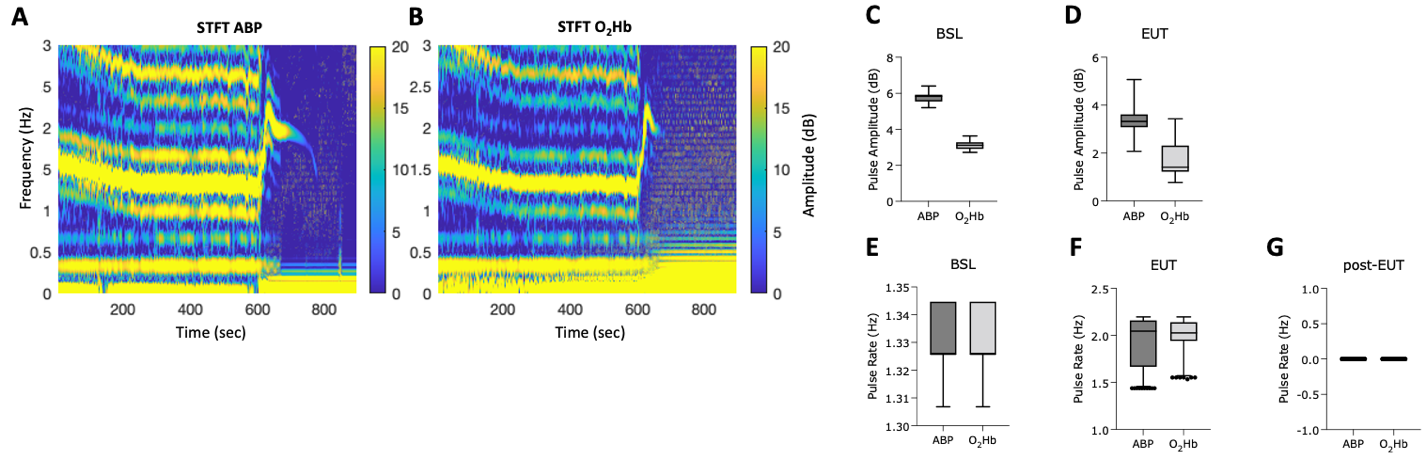


**Supplementary Figure S5** STFT analysis (animal #5) of **(A)** carotid ABP and (**B**) NIRS O_2_Hb signals over a 15-minute window. (**C-D**) Differences in the mean and variance of ABP and O_2_Hb-derived pulse amplitudes (dB) and (**E-G**) pulse rates (Hz) at baseline, during- and post-euthanasia. O_2_Hb -oxygenated hemoglobin; ABP – arterial blood pressure; BSL – baseline; EUT – euthanasia.

**STFT analysis (animal #6)**


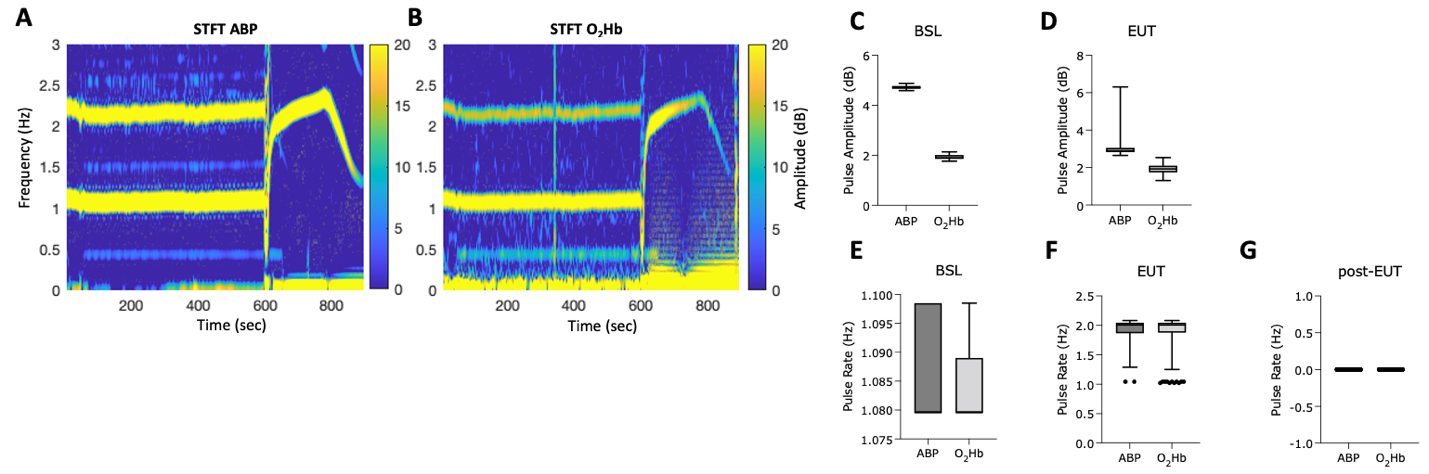


**Supplementary Figure S6** STFT analysis (animal #6) of **(A)** carotid ABP and (**B**) NIRS O_2_Hb signals over a 15-minute window. (**C-D**) Differences in the mean and variance of ABP and O_2_Hb-derived pulse amplitudes (dB) and (**E-G**) pulse rates (Hz) at baseline, during- and post-euthanasia. O_2_Hb -oxygenated hemoglobin; ABP – arterial blood pressure; BSL – baseline; EUT – euthanasia.

**STFT analysis (animal #7)**

**
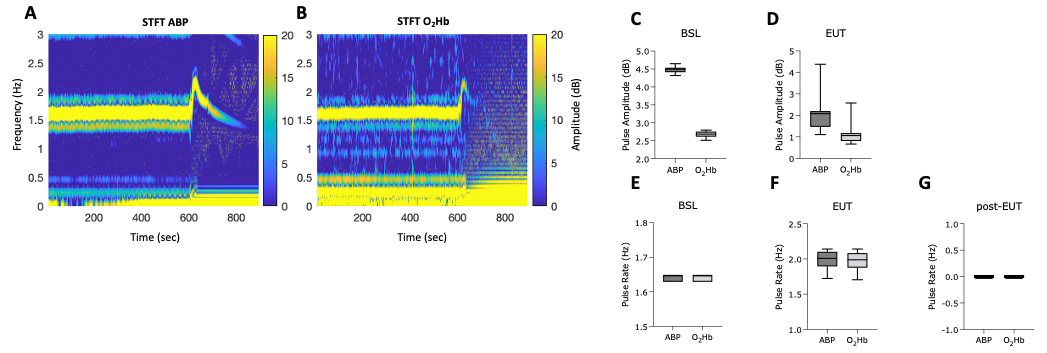
**

**Supplementary Figure S7** STFT analysis (animal #7) of **(A)** carotid ABP and (**B**) NIRS O_2_Hb signals over a 15-minute window. (**C-D**) Differences in the mean and variance of ABP and O_2_Hb-derived pulse amplitudes (dB) and (**E-G**) pulse rates (Hz) at baseline, during- and post-euthanasia. O_2_Hb -oxygenated hemoglobin; ABP – arterial blood pressure; BSL – baseline; EUT – euthanasia.

**STFT analysis (animal #8)**


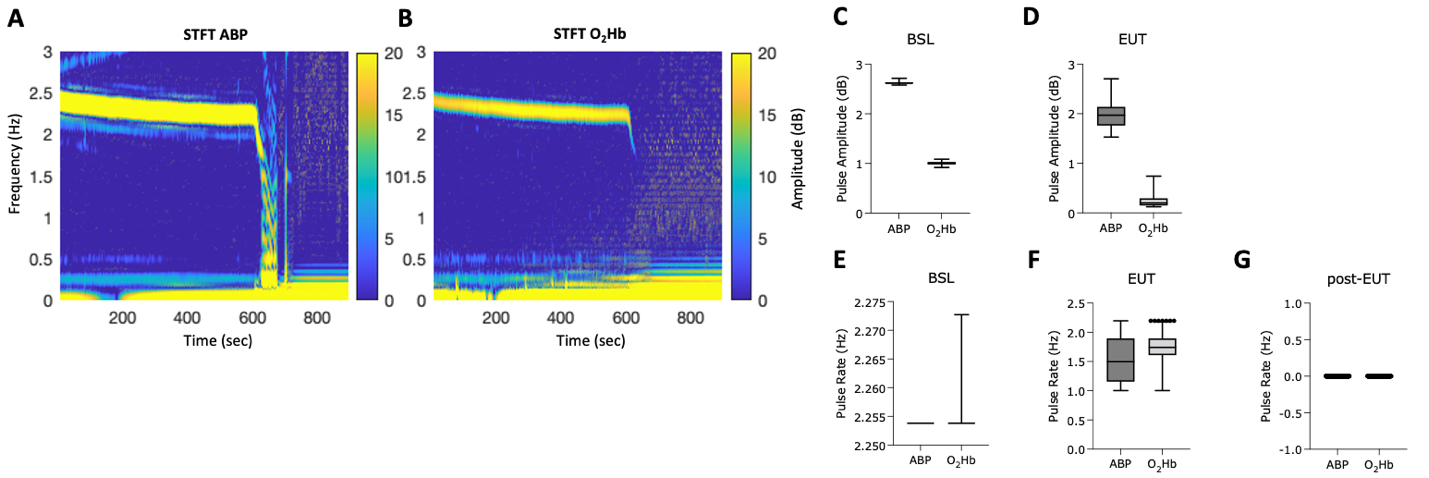


**Supplementary Figure S8** STFT analysis (animal #8) of **(A)** carotid ABP and (**B**) NIRS O_2_Hb signals over a 15-minute window. (**C-D**) Differences in the mean and variance of ABP and O_2_Hb-derived pulse amplitudes (dB) and (**E-G**) pulse rates (Hz) at baseline, during- and post-euthanasia. O_2_Hb -oxygenated hemoglobin; ABP – arterial blood pressure; BSL – baseline; EUT – euthanasia.
